# Systemic versus local delivery of mesenchymal stem cells to improve the early stages of fracture healing in a polytrauma model

**DOI:** 10.1186/s13036-025-00554-4

**Published:** 2025-09-30

**Authors:** Augustine Mark Saiz, Maryam Rahmati, Soren David Johnson, Aneesh Satish Bhat, Tony Daniel Baldini, Øystein Øvrebø, Liebert Parreiras Nogueira, Thaqif El Khassawna, Sabine Stötzel, Fernando A. Fierro, Mark A. Lee, J. Kent Leach, Håvard Jostein Haugen

**Affiliations:** 1 Department of Orthopaedic Surgery, UC Davis Health, 4860 Y Street, Suite 3800, Sacramento, CA 95817 USA; 2 Department of Biomaterials, Institute of Clinical Dentistry, University of Oslo, Oslo, 0318 Norway; 3 Oral Research Laboratory, Institute of Clinical Dentistry, University of Oslo, Oslo, 0318 Norway; 4 Experimental Trauma Surgery, Justus-Liebig University Giessen, Giessen, Germany; 5 Faculty of Pharmacy, University of Jordan, Amman, Jordan; 6 Institute for Regenerative Cures, University of California Davis, 2921 Stockton Blvd. Room 1300, Sacramento, CA 95817 USA; 7 Department of Cell Biology and Human Anatomy, University of California Davis, Davis, USA

**Keywords:** Inflammation, Polytrauma, Bone fracture, Mesenchymal stem cell, Hydrogel

## Abstract

Fracture healing complications, including nonunion, are common in polytrauma patients partly due to systemic inflammatory dysregulation. Although mesenchymal stem cells (MSCs) have been widely explored for their regenerative properties, their therapeutic potential in polytrauma patients remains uncertain. Given the clinical interest in both systemic and localized stem cell therapies, understanding how delivery route influences MSCs biodistribution, inflammatory modulation, and therapeutic efficacy is critical for optimizing treatment strategies in polytrauma. Hence, we compared systemic versus local MSCs delivery in a polytrauma model. We evaluated inflammatory responses and bone formation in a C57BL/6J murine model across four groups: (1) isolated fracture, (2) polytrauma (femur fracture + chest trauma), (3) polytrauma + systemic MSC delivery, and (4) polytrauma + local MSC delivery in hyaluronic acid-based hydrogels at the fracture site. Polytrauma induced a prolonged inflammatory response characterized by elevated interleukin 1 alpha and beta (IL-1α & β), tumor necrosis factor-alpha (TNF-α), interferon-gamma (IFN-γ), and monocyte chemoattractant protein-1 and − 5 (MCP-1 & MCP-5). Both delivery methods significantly reduced inflammation and proinflammatory cytokines, though local delivery yielded more consistent effects. IVIS imaging confirmed MSC retention at the fracture site in the local delivery group, while systemic administration of MSCs resulted in pulmonary entrapment. Although systemic MSCs failed to enhance fracture healing significantly, local MSC delivery promoted bone formation evidenced by CT and histological characterizations. These findings demonstrate that local MSC delivery in a hydrogel scaffold represents a superior strategy for improving fracture healing in polytrauma patients compared to systemic delivery.

**Supplementary Information:**

The online version contains supplementary material available at 10.1186/s13036-025-00554-4.

## Introduction

Effective fracture healing is essential for restoring skeletal integrity and function [1, 2]. If the fractured bones fail to heal properly, called nonunion, patients suffer from chronic pain and disability [1, 2]. Understanding the factors that disrupt normal bone repair is crucial for developing strategies to prevent and treat nonunion [3]. Polytrauma, defined as multiple simultaneous injuries affecting different body regions or systems, adversely affects fracture healing. For instance, one study demonstrated that polytrauma impairs fracture healing, accompanied by increased systemic inflammation and immune dysregulation [4]. Chest and pulmonary injuries are common in polytrauma cases [2, 4], and these thoracic injuries can worsen systemic inflammation, hindering bone repair [2, 4]. ​In our previous study [2], we investigated the impact of polytrauma on fracture healing using a murine model combining femur fracture with chest trauma [2]. We observed that polytrauma led to a dysregulated inflammatory response characterized by a persistent increase in innate immune cells at the fracture site and systemically coupled with a significant reduction in adaptive immune cell populations. This immune imbalance was associated with impaired fracture healing, evidenced by forming a poorly mineralized and disorganized fracture callus. Together, these findings underscore how immune dysregulation in polytrauma hinders bone repair [2].

Despite surgical advancements, few therapies effectively manage fracture healing in polytrauma patients. While current biologic materials, such as bone morphogenetic proteins (BMPs) on collagen sponges or demineralized-based matrix (DBM)-based products, are primarily utilized in nonunion repair, these strategies are not designed to address the systemic immune dysregulation observed in polytrauma. This limitation reduces their effectiveness in acute multisystem injury settings, where a prolonged inflammatory response can impede bone healing. For instance, studies have indicated that BMP-2 delivered via collagen sponges may correlate with systemic immune dysregulation, highlighting the need for systems that can modulate inflammatory responses and promote osteogenesis [15]. Therefore, innovative approaches that address both the inflammatory and osteogenic components of healing are needed.

Mesenchymal stem cells (MSCs) have emerged as a promising therapeutic approach for modulating inflammation and promoting tissue regeneration [5, 6]. Their ability to hone to injury sites and secrete anti-inflammatory cytokines makes them attractive for treating inflammatory conditions [5, 6]. However, the route of MSC administration significantly influences their distribution and efficacy. Systemic administration often results in pulmonary entrapment due to the pulmonary first-pass effect, preventing MSCs from reaching the fracture site [7]. For instance, Eggenhofer et al. [8] demonstrated that MSCs are primarily trapped in the lungs following intravenous injections and do not migrate beyond this point. This entrapment leads to a short lifespan of the MSCs, as they do not survive long after being sequestered in the pulmonary system [8]. This phenomenon presents a major challenge, particularly in polytrauma settings, where systemic inflammation and competing injury signals further hinder MSC homing and survival. To overcome such limitations, local MSC delivery using biomaterial scaffolds has been explored as an alternative strategy [9, 10]. Hyaluronic acid (HA)-based hydrogels offer a promising solution by providing a biocompatible, degradable, and bioactive environment that enhances MSC retention at the injury site. HA, a natural extracellular matrix (ECM) component, supports cell adhesion, hydration, and remodeling [11]. Its hydrophilic structure ensures MSC viability while preventing rapid cell clearance, addressing one of the key drawbacks of systemic delivery. Additionally, HA hydrogels can be engineered to control MSC release over time, allowing for sustained therapeutic effects and a more gradual, regulated immune response [12]. Beyond its physical scaffold properties, HA also exhibits immunomodulatory effects, reducing excessive macrophage activation and mitigating prolonged inflammatory responses in injured tissues [13]. Given these advantages, HA-based hydrogels represent a clinically relevant approach for MSC-based therapies, particularly in polytrauma associated fractures in the setting of immune dysregulation and impaired bone healing.

In this study, we employed a polytrauma model involving femur fracture and chest trauma to (1) compare the inflammatory response in polytrauma versus isolated fracture, and (2) evaluate the effects of local versus systemic MSC delivery on fracture healing, particularly using HA-based hydrogels to enhance local retention and therapeutic impact. We hypothesized that the direct implantation of MSCs encapsulated in an HA-based hydrogel at the fracture gap circumvents pulmonary sequestration and restores callus formation, mineralization, and mechanical strength to levels achieved in isolated-fracture controls. By elucidating the inflammatory mechanisms underlying polytrauma-induced fracture healing impairment and assessing the efficacy of different MSC delivery methods, this study aims to inform therapeutic strategies for improving outcomes in patients with complex injuries.

## Materials and methods

### In vitro assays

#### Cell culture

Mouse bone marrow-derived MSCs (AcceGen, Fairfield, NJ) were expanded on tissue culture plastic in growth media (GM) composed of alpha-MEM supplemented with 10% fetal bovine serum (GenClone, Genesee Scientific, San Diego, CA) and 1% penicillin-streptomycin (Gemini Bio Products, West Sacramento, CA), under standard conditions. For osteogenic differentiation, GM was supplemented with 0.01 µM dexamethasone, 50 µg/mL L-ascorbic acid 2-phosphate, and 10 mM sodium β-glycerophosphate. Osteogenic medium was refreshed every 48 h. MSCs were used at passage 5 and seeded at 25,000 cells per well in 24-well plates (*N* = 8 per group) for 24 h prior to treatment.

#### Hydrogel synthesis

HA hydrogels were synthesized using pharmaceutical-grade 3 MDa hyaluronic acid (H.T.L. SAS Javené, France), crosslinked with 1.6% v/v 1.4-butanediol diglycidyl ether (BDDE, Merck KGaA, Darmstadt, Germany) along with anhydrous sodium hydroxide and sodium chloride (Merck KGaA, Darmstadt, Germany). A 10 w/v% hyaluronic acid (HA) solution was prepared in 0.3 M NaOH under manual stirring. Subsequently, 1.6 v/v% BDDE, and the mixture was maintained at 40 °C for 4 h in a closed vessel. The resulting gel was placed in a cellulose membrane (molecular weight cutoff = 14 kDa) and dialyzed against water for injection (B.Braun, Melsungen AG, Melsungen, Germany) for 18 h. The gel was granulated by passing it through a 130 μm mesh after dialysis. Water for injection and 10 wt% sodium hyaluronate were added until a final HA concentration of 22 mg/mL was achieved. For detailed results regarding material characterization please refer to Øvrebø et al. [14]. or to the summary provided in the supplementary file of this manuscript.

#### Determination of cytokine profiling

To study inflammatory secretome responses of MSCs to isolated fracture versus polytrauma injuries in a controlled in vitro environment. After 24 h of culture, MSCs were treated for 48 h with GM supplemented with 10% pooled mouse serum (C57BL/6J; *N* = 8 per group) obtained from polytrauma or isolated fracture mice at 72 h post-injury. Media was then replaced with for further 3 or 7 days. Conditioned media was collected at each time point and analyzed using RayBiotech cytokine antibody arrays (C-Series 1000.1, RayBiotech, Inc., Norcross, GA) following manufacturer instructions.

#### Labeling mesenchymal stem cells with GFP and luciferase

GFP and luciferase-expressing MSCs were transduced as described previously in [15, 16]. MSCs were transduced using a third-generation lentiviral vector (pCCLc-MNDU3-Luciferase-PGK-eGFP-WPRE) with protamine sulfate (20 µg/mL). Transduction efficiency was ~ 95% GFP + at 72 h.

#### Animal surgery

We performed animal surgeries in the Department of Orthopaedic Surgery, University of California, Davis in compliance with the ARRIVE guidelines under an approved protocol from the UC Davis Institutional Animal Care and Use Committee (IACUC# 23193). We acquired a total of 96 10-week-old male C57BL/6J mice (Jackson Laboratories, Bar Harbor, ME). Mice were randomly housed in groups of four and were acclimated to the housing vivarium for two weeks prior to any procedures. Water and pellet diet were provided *ad libitum*. At 12 weeks of age, animals were randomly divided into four groups: (1) Isolated Fracture, (2) Polytrauma, (3) Polytrauma + Sys MSCs (systemic delivery of MSCs in polytrauma mice) and 3) Polytrauma + Local MSCs (Local delivery of MSCs at the fracture site of polytrauma mice). Mice were injected subcutaneously with buprenorphine (0.1 mg/kg) for pain control and saline 5–10 min before surgery and weighed. Femur fracture and chest injuries were induced according to our previous study [2]. Briefly, for the femur fracture, mice were anesthetized with 2–4% isoflurane in oxygen and the right hind-limb was shaved and prepped in a standard sterile manner. An incision was made over the anterior knee joint, and the distal femur was exposed. The knee was flexed, and the right femur was reamed through the femoral condyles with a 30G needle as an intramedullary pin (IM). The joint capsule was closed using Monocryl (Johnson & Johnson, Brunswick, NJ) sutures and the skin closed with nylon sutures. Using an Einhorn drop weight device, we created a transverse femur fracture. X-ray imaging confirmed the correct placement of the needle and the induced fracture. The fracture apparatus has been described elsewhere [17, 18].

After fracture and while still under anesthesia, we induced blunt thoracic trauma by dropping a hollow aluminum cylindrical weight (~ 30 g) from a height of 55 cm through a vertical stainless-steel tube onto a Lexon platform resting on the animal’s chest [2]. This apparatus has been described and characterized for this application elsewhere [19].

MSCs were then delivered systemically or locally. Systemic delivery: 1 × 10⁶ GFP-luciferase-labeled MSCs in GM were injected into the lateral tail vein using a 27G needle. Local delivery: MSCs were encapsulated in 80 µL HA hydrogel and injected directly at the fracture site. Mice were allowed to recover from anesthesia in a well-ventilated area and kept warm using a heat pad underneath half of the cage with a surgical towel to minimize risk of contact thermal injury. Animal were observed for 10 d after surgery. Mice were injected with buprenorphine (0.1 mg/kg) twice daily after surgery (approximately 12 h apart) for 48 h. Mice were also weighed to ensure that no more than 20% of weight was lost from baseline weight. The surgical site was examined daily until the sutures were removed or the animals were euthanized. The animals were full weight-bearing and unrestricted activity was permitted postoperatively.

Mice were euthanized by exsanguination *via* cardiac puncture under anesthesia, followed by cervical dislocation. Blood was collected during cardiac puncture to analyze cytokines within the serum. Femur tissues were dissected and fixated in 4% paraformaldehyde for the downstream fracture healing analyses. For the in vivo aspect of this work, we only included the treatment groups as in our previous study [2], we characterized the healing outcomes in both isolated fracture and polytrauma groups without any therapeutic intervention. That work demonstrated that polytrauma significantly impairs fracture healing compared to isolated fracture, establishing a baseline for understanding the impact of systemic injury on bone repair. To adhere to the principles of the 3Rs (Replacement, Reduction, and Refinement) in animal research and avoid unnecessary duplication, we did not include untreated control groups in the present study. Instead, we focused on comparing the effects of systemic versus local MSC delivery in polytrauma conditions, building directly upon our previously published data. By referencing these established baseline outcomes, we aimed to reduce animal usage while still enabling meaningful interpretation of therapeutic efficacy.

### Analysis of cytokines within serum

Whole blood collected *via* cardiac puncture was processed for serum cytokine (biological replicates, *N* = 8, technical replicates, *N* = 2). Sample preparation followed the manufacturer’s protocol (Biolegend, San Diego, CA) and analyzed using a BD Fortessa flow cytometer at the Institute of Regenerative Medicine, University of California, Davis. Data analysis was performed using Biolegend software for cytokine analysis (Biolegend, San Diego, CA).

### In vivo imaging system (IVIS) imaging

IVIS-imaging was conducted on days 3, 7 and 21, post-delivery of labeled MSCs. Mice were injected intraperitoneally with D-luciferin (150 mg/kg, GoldBio, St. Louis, MO) 10–15 min before imaging. Bioluminescence was captured in 2–4% isoflurane-anesthetized mice using an IVIS Spectrum System (PerkinElmer, Waltham, MA) with a 1-minute exposure and 8 × 8 binning. Images were analyzed using Aura Living Image Software (PerkinElmer).

### 3D micro-computed tomography analysis

We imaged femur samples (fixed in 4% paraformaldehyde, 6 mice per treatment group, totaling 12 mice) using a microCT specimen scanner (Bruker microCT2242, Kontich, Belgium). Scan parameters were 55 kVp, 145 µA, 300 millisecond exposure time, average of 3 exposures per projection, 0.5 mm aluminum filter, 500 projections per 180 degrees and a 10 μm nominal voxel size. The raw images were calibrated using a hydroxyapatite (HA) phantom of varying HA concentrations. Noise in the images was reduced using a low-pass Gaussian filter. A 1 mm region of interest (ROI) extending from the defect edge was analyzed to capture bone outgrowth and account for anatomical variation between mice. Bone volume fraction (BV/TV) was determined by dividing the number of voxels denser than the low threshold representing mineralized tissue (BV: bone volume) by the total number of pixels in the region (TV: total volume). Several other parameters, including bone surface density (BS/TV), trabecular thickness (Tb.Th), trabecular separation (Tb.Sp), fractal dimension and degree of anisotropy were also measured.

### Histological and immunohistochemical analysis

After microCT imaging, we performed decalcified histology on the samples (6 mice per group, a total of 12 mice). As described in our previous papers, Movat Pentachrome was used to evaluate the balance between bone mineralization and non-mineralization over time. Movat Pentachrome stain was used to image different constituents of the connective tissue: mineralized bone appears bright yellow, cartilage appears as blue-green, and non-mineralized bone appears bright red [20, 21]. Alkaline Phosphatases (ALP) was performed to evaluate the osteoblast activity [20, 21]. The immunohistochemistry analysis was performed using primary antibodies (Abcam Company, Cambridge, UK). The following antibodies were used: Anti-RANKL antibody (ab216484), anti-osteoprotegerin antibody (ab73400), and anti-factor VIII antibody (ab275376). For more details on these assays readers can refer to [20, 21].

### Quantitative histomorphometrical analysis

Sections were imaged at 10 × (3.09 pixels/µm) magnification using a Leica microscopy system (Leica DM5500 photomicroscope equipped with a DFC7000 camera and operated by LASX software version 3.0, Leica Microsystems Ltd, Wetzlar, Germany). Fiji ImageJ was used for histomorphometry. Fiji ImageJ (version 1.51r; NIH, Maryland, USA) was used as a platform to run the program. The Trainable Weka Segmentation (TWS) was used as the basis for creating an optimized script to analyze tissue formation parameters, including mineralization, new bone (non-mineralized bone), and cartilage formation. The histomorphometry measurements were done as previously reported by Malhan et al. [22].

### Statistical analysis

Statistical analysis was performed using Prism 9.5.1 (GraphPad, San Diego, CA). Data normality was assessed using the Kolmogorov-Smirnov test. Normally distributed data are presented as mean ± standard deviation (SD), while non-normally distributed data are reported as median with interquartile range (IQR). When data failed the normality test, non-parametric analysis was performed using the Kruskal-Wallis test or one- /two-way ANOVA, depending on the experiment. For normally distributed datasets, multi-factor regression analysis was performed. Significant differences were denoted as *P < 0.05, **P < 0.01, ***P < 0.001 and ****P < 0.0001.

## Results

### Polytrauma serum enhances Proinflammatory cytokine and chemokine secretion from MSCs compared to fracture serum at day 3

To investigate how MSCs respond differently to fracture versus polytrauma environments, we performed secretome profiling targeting a broad range of cytokines and chemokines at days 3 and 7 post-culture. The results revealed distinct inflammatory and regenerative profiles between the two conditions, underscoring the immunological challenges posed by polytrauma.

**Fig. 1:**
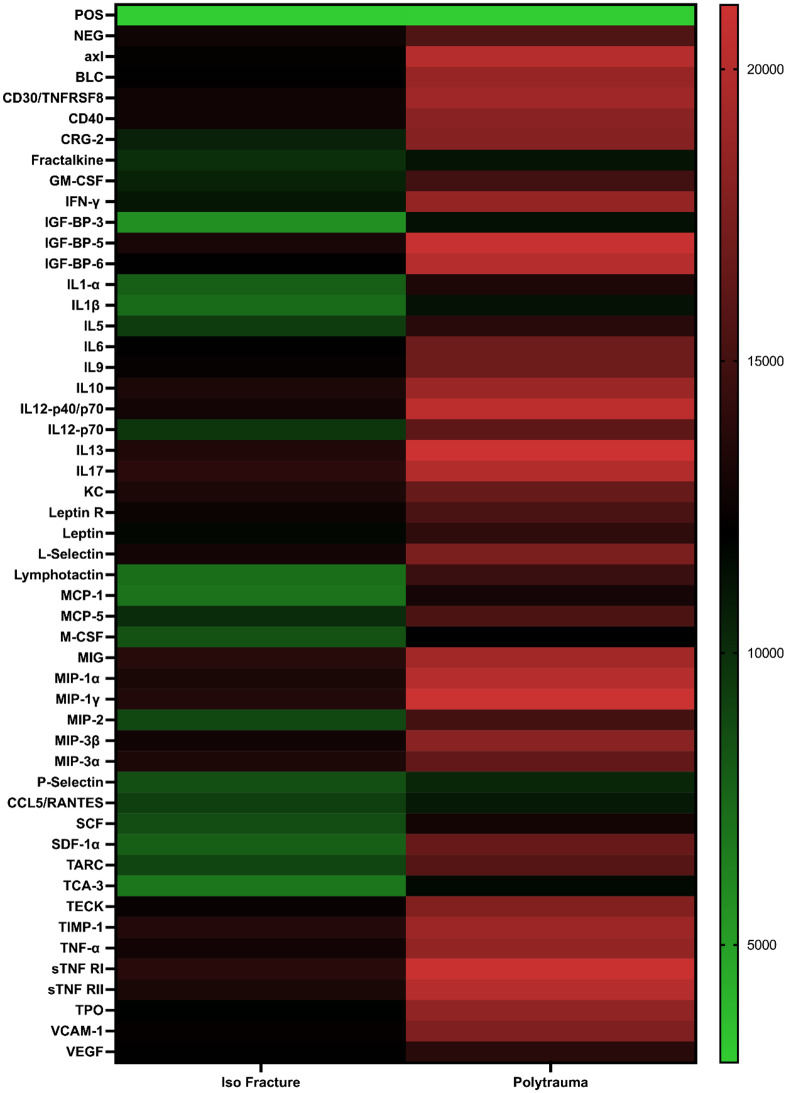
Polytrauma serum induces higher proinflammatory responses in mesenchymal stem cells (MSCs). Secretome profiling of mesenchymal stem cells (MSCs) pre-exposed to isolated fracture versus polytrauma serum after 3 days of culturing in osteogenic media. Cytokine levels were quantified, highlighting differential inflammatory responses between the two conditions. *N* = 8, samples were pooled together for each group. Green color depicts low levels and red depicts high levels of expression. POS = positive control spots, which was used as the internal control/baseline

As shown in Fig. 1 and Supplementary Fig. 1, MSCs cultured in polytrauma serum exhibited significantly elevated levels of multiple pro-inflammatory cytokines after three days in osteogenic media. These included interleukins IL-6, IL-1α, IL-1β, IL-5, IL-9, IL-12p70, IL-13, IL-15, and IL-17. Increased levels of tumor necrosis factor-alpha (TNF-α), its soluble receptors sTNF-RI and sTNF-RII, interferon-gamma (IFN-γ), and granulocyte-macrophage colony-stimulating factor (GM-CSF) were also observed. Collectively, these cytokines are known to recruit and activate inflammatory cells, potentially amplifying the inflammatory response at injury sites.

Additionally, MSCs exposed to polytrauma serum secreted higher levels of chemokines, including monocyte chemoattractant proteins (MCP-1 and MCP-5), and macrophage inflammatory proteins (MIP-1α, MIP-1γ, MIP-2, MIP-3α, and MIP-3β).

In contrast, fracture serum elicited a more balanced inflammatory response, with lower levels of these pro-inflammatory factors. Instead, it was associated with higher expression of vascular and regenerative mediators including vascular cell adhesion molecule-1 (VCAM-1), and vascular endothelial growth factor receptor 1 (VEGF-R1) reflecting a more pro-angiogenic and tissue -repair–oriented secretome.

.

### Polytrauma serum sustains a highly inflammatory MSC secretome at day 7

**Fig. 2:**
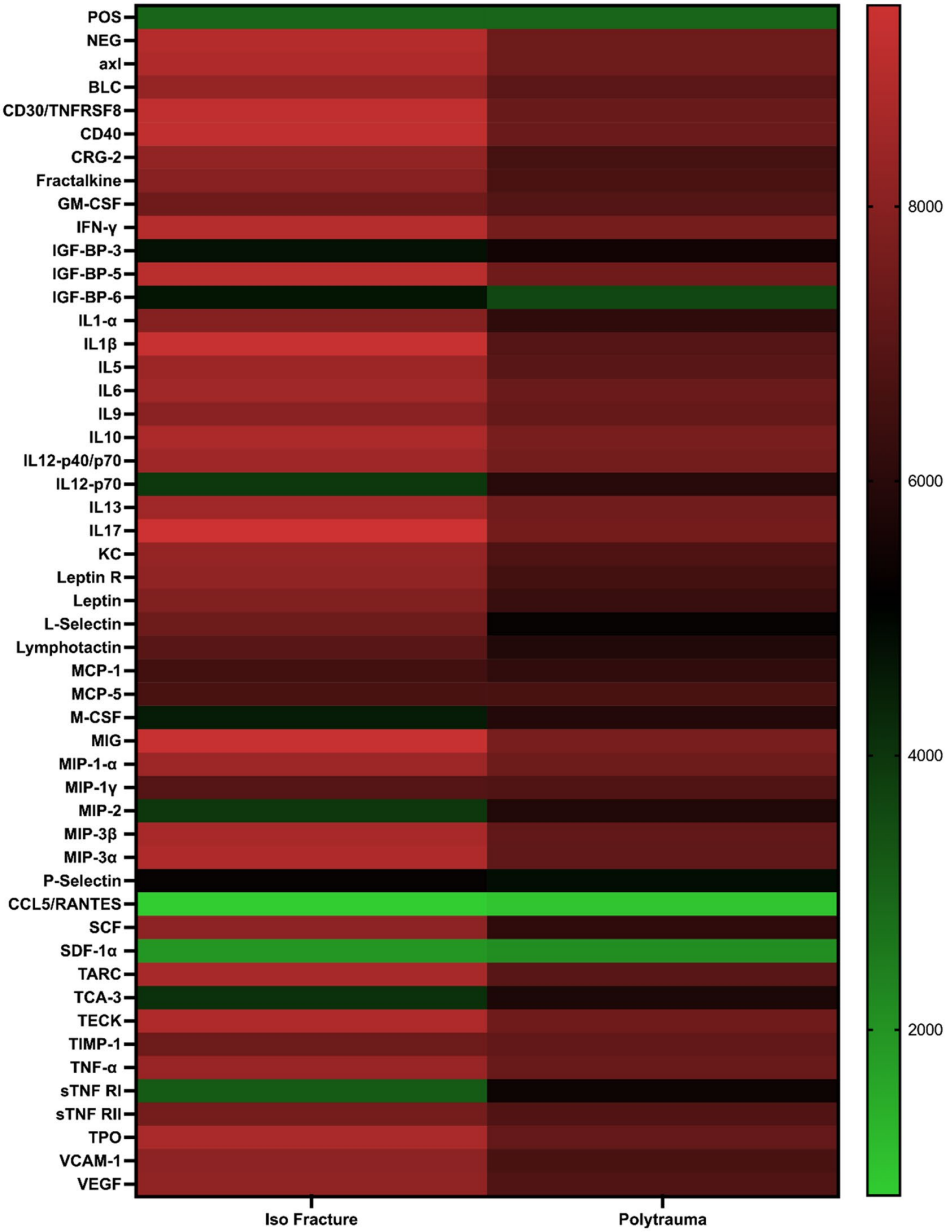
Polytrauma serum induces higher proinflammatory responses in mesenchymal stem cells (MSCs). Secretome profiling of MSCs pre-exposed to isolated fracture versus polytrauma serum after 7 days of culturing in osteogenic media. Cytokine levels were quantified, highlighting differential inflammatory responses between the two conditions. *N* = 8, samples were pooled together for each group. POS = positive control spots, which was used as the internal control/baseline

By day 7 of osteogenic culture, MSCs exposed to polytrauma serum continued to secrete elevated levels of pro-inflammatory cytokines compared to those cultured with fracture serum (Fig. 2 and Supplementary Fig. 2), however this difference was not statistically significant. Interleukins including IL-6, IL-1α, IL-1β, IL-5, IL-9, IL-12p70, IL-13, IL-15, and IL-17 remained elevated, alongside sustained expression of TNF-α, its soluble receptors (sTNF-RI and sTNF-RII), IFN-γ, and GM-CSF.

The chemokine profile remained similarly pro-inflammatory. MSCs exposed to polytrauma serum showed increased secretion of MCP-1, MCP-3, MCP-5, and MIP-1α, MIP-1γ, and MIP-3β. Notably, CXCL15 (Lungkine), a lung-associated chemokine, was upregulated, further distinguishing the polytrauma response.

Conversely, fracture serum-exposed MSCs showed increased expression of anti-inflammatory cytokines such as IL-10 and angiogenesis-promoting factors like VCAM-1, AND VEGF-R1.

Together, these findings demonstrate that MSCs exposed to polytrauma serum sustain a pro-inflammatory secretome. In contrast, fracture serum has a higher likelihood of promoting an anti-inflammatory environment.

### Polytrauma triggers sustained systemic inflammation, which is attenuated by MSC therapy

**Fig. 3:**
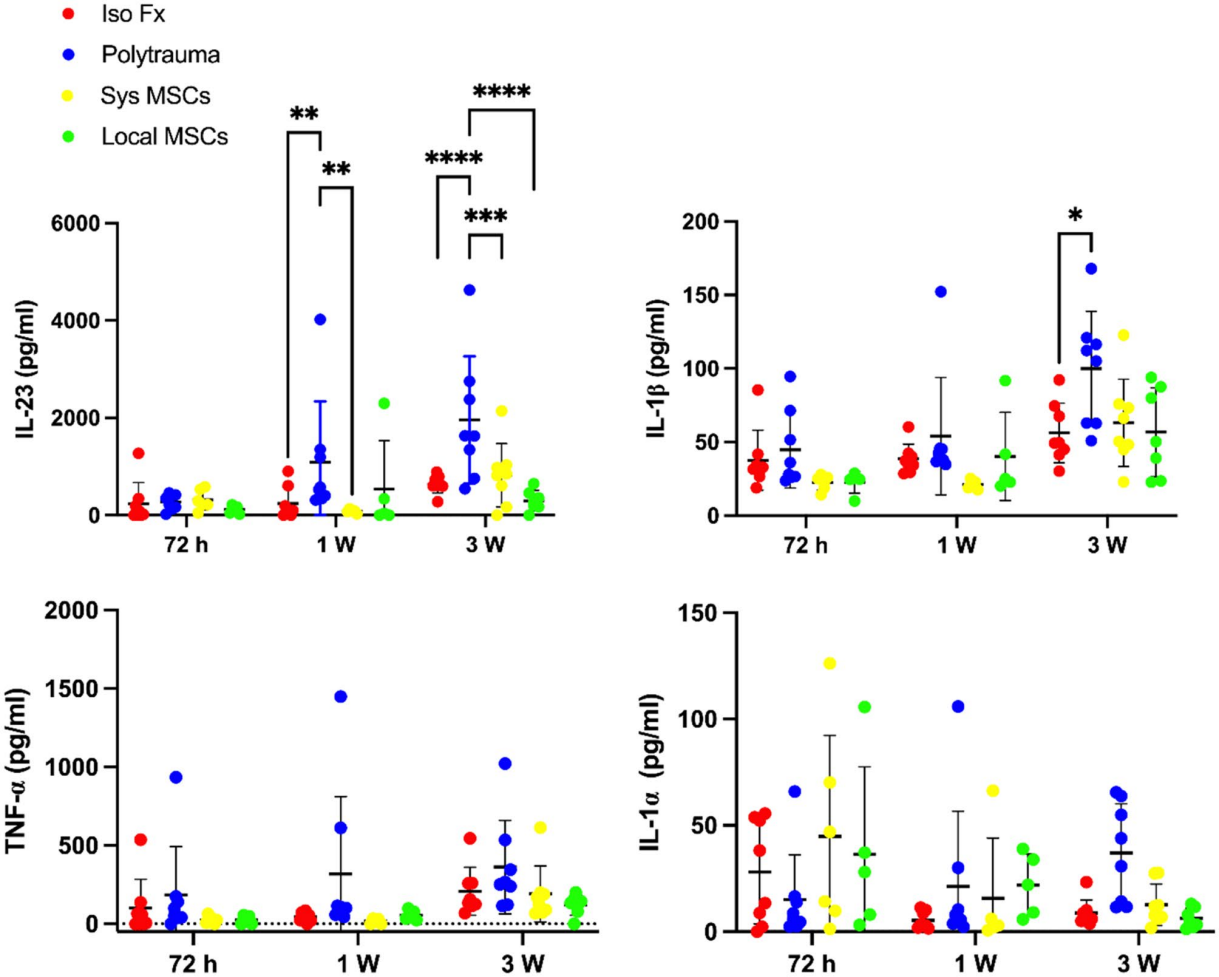
Mesenchymal stem cells (MSCs) attenuate inflammatory response in polytrauma environments. Cytokine expression of polytrauma mice treated with systemic or local delivery of MSC. Levels of inflammatory cytokines (interleukin 23, IL-23, interleukin 1 beta, IL-1β, tumor necrosis factor alpha, TNF-α, and interleukin 1 alpha, IL-1α) were measured from serum samples, revealing differences in systemic inflammatory responses between treatment groups. *N* = 8. Data presented as mean ± SD. Significant differences between groups were presented. Significant differences were presented as *P < 0.05, **P < 0.01, ***P < 0.001 and ****P < 0.0001

Polytrauma provoked a markedly heightened systemic inflammatory response compared to isolated fracture, as reflected by elevated serum levels of key cytokines including IL-23 at both 1- and 3-weeks post-injury, and IL-1β at 3 weeks (Fig. 3). Increases in TNF-α and IL-1α were also observed, albeit to a lesser extent, suggesting broad activation of inflammatory signaling. This amplified cytokine profile underscores the physiological complexity and systemic disruption characteristic of polytrauma.

Importantly, both systemic and local MSC delivery significantly mitigated these inflammatory responses. Local MSC therapy led to a greater reduction in IL-23 levels at both time points. Although differences in TNF-α level did not reach statistical significance, a downward trend was evident, particularly in the locally treated group. Notably, MSC treatment, especially *via* local delivery, also reduced the variability in cytokine levels across individuals, suggesting a stabilizing effect on systemic immune dysregulation.

These findings emphasize that MSC therapy can help temper the hyperinflammatory state induced by polytrauma, with local delivery offering potentially more consistent modulation of systemic cytokine responses.

### MSC delivery suppresses type I interferons and IL-12p70, with local delivery providing yielding a uniform Immunomodulation

**Fig. 4:**
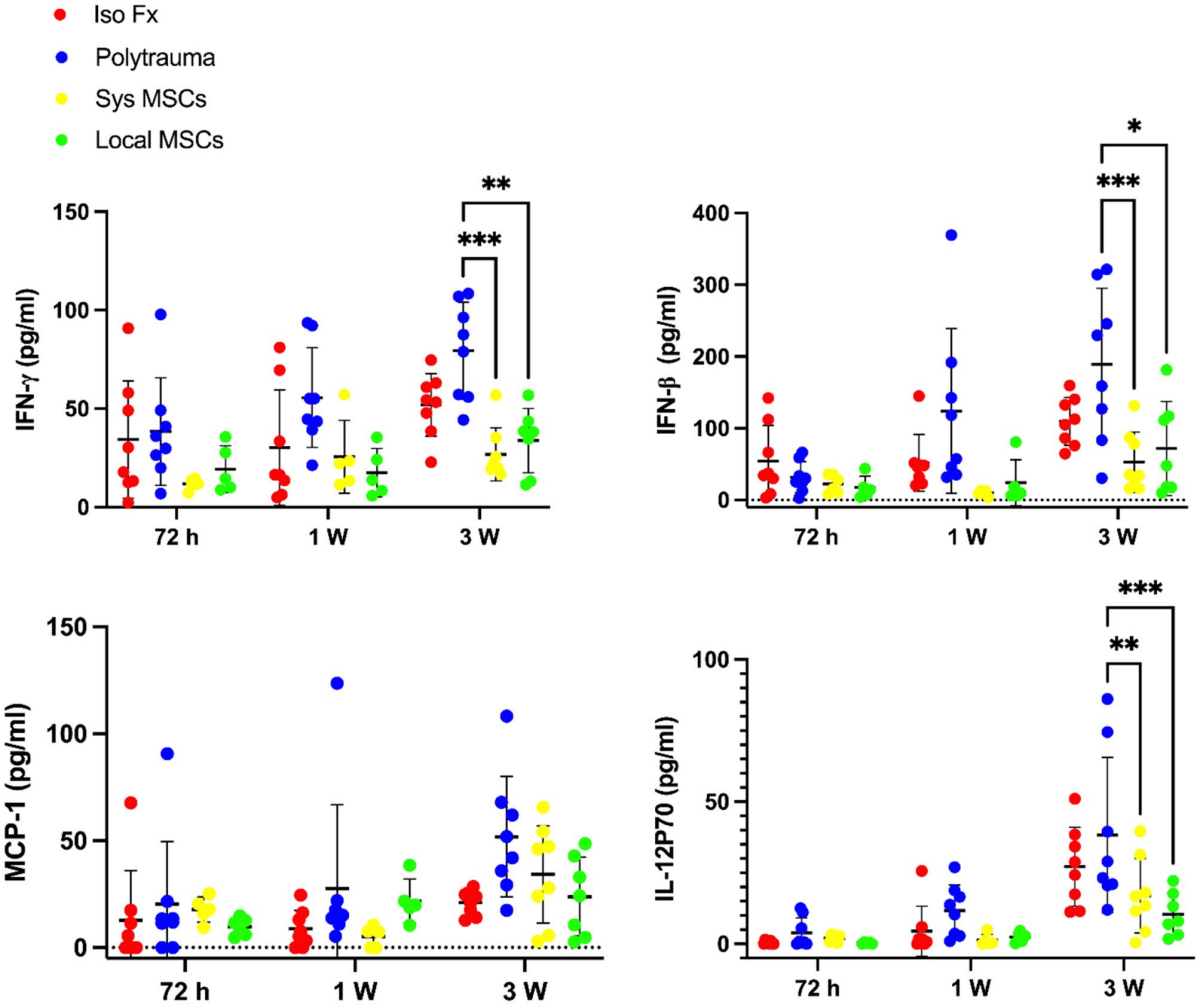
Mesenchymal stem cells (MSCs) attenuate inflammatory response in polytrauma environments. Cytokine expression of polytrauma mice treated with systemic or local delivery of MSC. Levels of inflammatory cytokines (interferon gamma, IFN-Y, interferon beta, IFN-β, monocyte chemoattractant protein 1, MCP-1, and interleukin 12P70, IL-12P70) were measured from serum samples, revealing differences in systemic inflammatory responses between treatment groups. *N* = 8. Data presented as mean ± SD. Significant differences between groups were presented. Significant differences were presented as *P < 0.05, **P < 0.01, and ***P < 0.001

Polytrauma induced elevated serum levels of IFN-γ, IFN-β, and IL-12p70 after 1- and 3-weeks post injury compared to the isolated fracture group, reflecting an intensified and prolonged systemic inflammatory response (Fig. 4). Notably, the polytrauma group also exhibited a significantly higher variability in cytokine and chemokine levels compared to isolated fracture, indicating a more heterogeneous and unpredictable immune response. The level of MCP-1 did not reach statistical significance.

Both systemic and local MSC delivery mitigated the elevated cytokine and chemokine levels in polytrauma mice sera. Significant differences between untreated and treated polytrauma groups were observed in IFN-γ, IFN-β, and IL-12p70 after 3 weeks of healing. While local MSC delivery showed a more robust reduction in IL-12p70, which plays a central role in sustaining inflammation [23, 24], systemic delivery of MSCs reduced the levels of IFN-γ, IFN-β more significantly. Importantly, local MSC delivery also dampened the variability in cytokine and chemokine observed in the polytrauma group. This more uniform immune response may enhance therapeutic predictability, especially in the context of complex injury scenarios where inflammatory responses are typically heterogeneous and difficult to manage.

### Local and systemic MSC delivery suppress chronic inflammatory cytokines, but only local delivery normalizes GM-CSF and IL-27

Key pro-inflammatory cytokines, including GM-CSF and IL-17 A, were increased in the polytrauma group after 3 weeks. These cytokines are associated with chronic inflammation and delayed tissue repair [25, 26]. Anti-inflammatory cytokine, IL-10, was comparatively lower in the polytrauma group after 3 weeks, suggesting an insufficient resolution of inflammation.

**Fig. 5:**
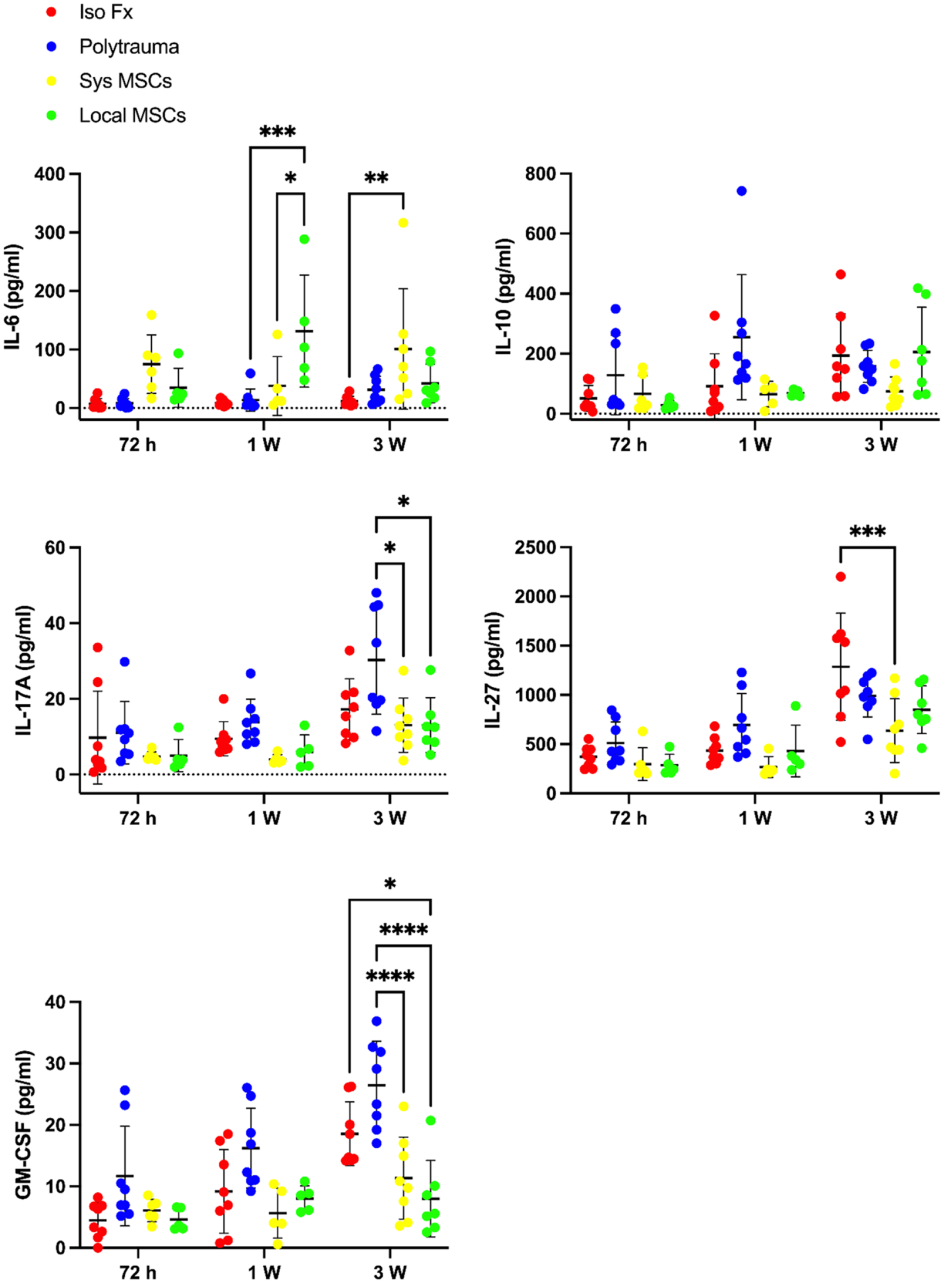
Mesenchymal stem cells (MSCs) attenuate inflammatory response in polytrauma environments. Cytokine expression of polytrauma mice treated with systemic or local delivery of MSC. Levels of inflammatory cytokines (interleukin 6, IL-6, interleukin 10, IL-10, interleukin 17-A, IL-17 A, interleukin 27, IL-27, and granulocyte-macrophage colony-stimulating factor, GM-CSF) were measured from serum samples, revealing differences in systemic inflammatory responses between treatment groups. *N* = 8. Data presented as mean ± SD. Significant differences between groups were presented. Significant differences were presented as *P < 0.05, **P < 0.01, and ***P < 0.001

Both systemic and local MSC delivery significantly reduced the inflammatory response in polytrauma mice by decreasing IL-17 A and GM-CSF after 3 weeks of healing. Systemic delivery of MSCs also significantly reduced the level of IL-27. Notably, the level of GM-CSF for the local delivery group was significantly lower than the isolated fracture group after 3 weeks. This trend was also seen for IL-27 after systemic delivery of MSCs. The expression of IL-6 was increased significantly in both local and systemic delivery of MSCs over time (Fig 5).

### Systemic MSCs are trapped in the pulmonary region, while locally delivered MSCs remain at the fracture site

**Fig. 6:**
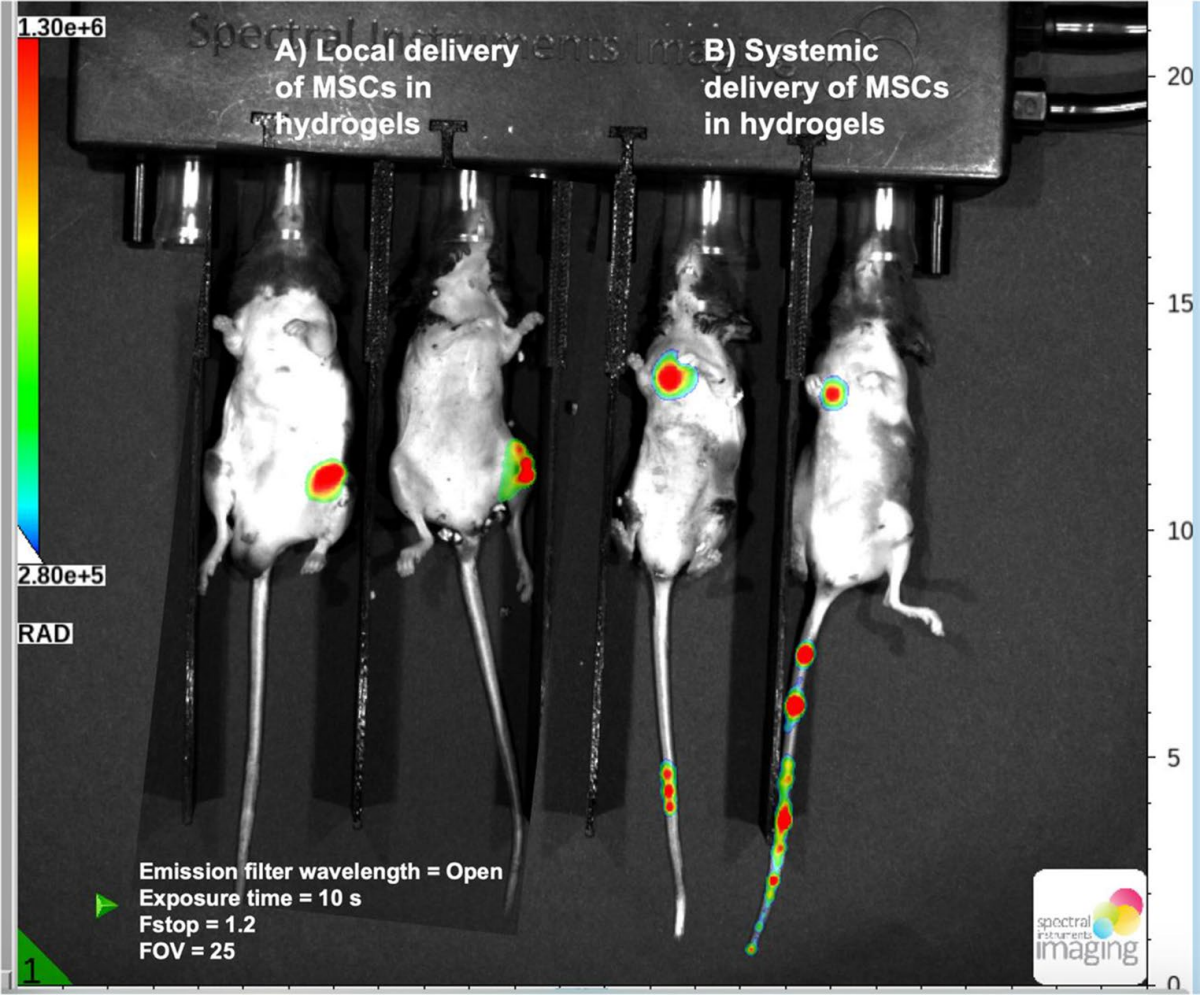
Mesenchymal stem cells (MSCs) delivered systemically in polytrauma mice are trapped in the lungs and did not travel to the fracture site. Representative in vivo imaging system (IVIS) images show luciferase activity from MSCs at the fracture site over time

As shown in Fig. 6, in vivo bioluminescent imaging (BLI) was used to visualize the location of luciferase-labeled MSCs one week post-delivery in polytrauma mice. Since the focus of this experiment was on the localization of MSCs rather than quantification, signal visualization was sufficient to evaluate the distribution of the cells. In the local delivery group, MSCs remained localized to the femur fracture site, as indicated by a strong bioluminescent signal in the region of the injury. This suggested that locally delivered MSCs were retained at the administration site, allowing them to interact with the injury microenvironment directly.

In the systemic delivery group, bioluminescent signals were predominantly observed in the pulmonary region, indicating that MSCs traveled to the lungs but did not reach the fracture site. The absence of detectable signals at the fracture site in the systemic group underscores that the majority of the MSCs were sequestered in the lungs and did not reach the site of injury.

### Local MSC delivery enhances bone formation and improves trabecular architecture compared to systemic MSC delivery

**Fig. 7:**
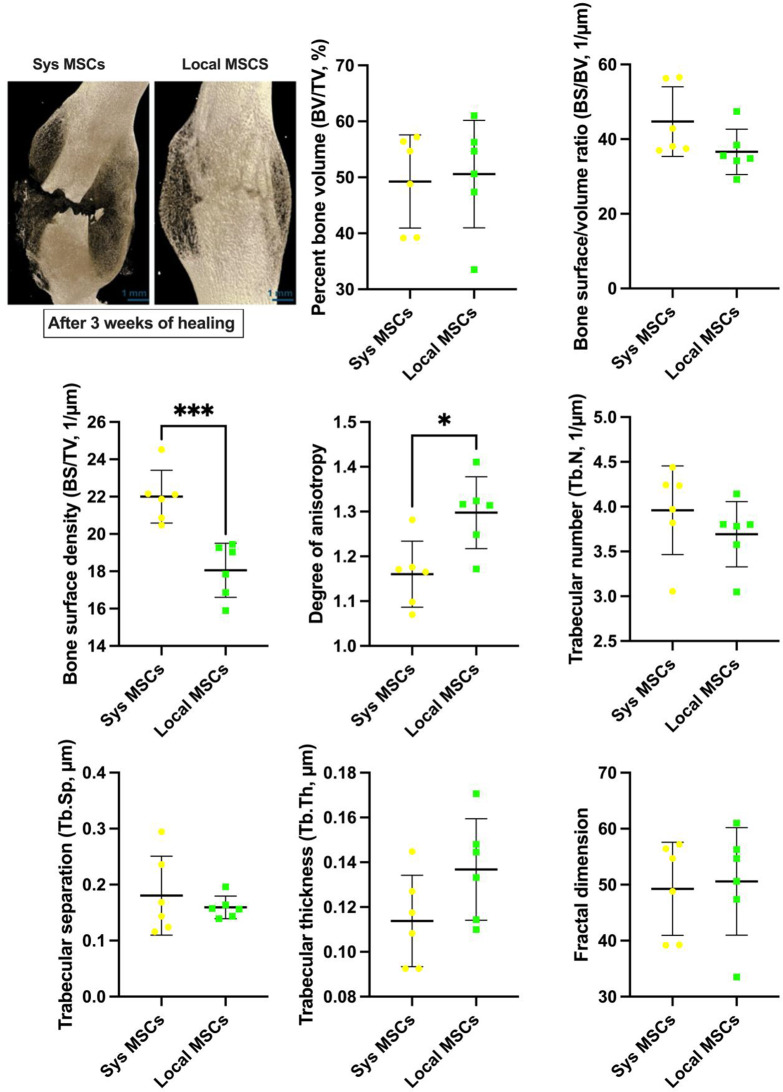
Locally injected mesenchymal stem cells (MSCs) enhance fracture healing in polytrauma after three weeks of healing. Top left panel) Representative reconstructed microCT images of the femur in the two groups of systemic MSCs (Sys MSCs) and Local MSCs delivered in hydrogels after three weeks of healing. Quantitative microCT analysis of bone volume and surface characteristics including bone volume fraction (BV/TV), bone surface/volume ratio (BS/BV), bone surface density (BS/TV), and degree of anisotropy (DA) as well as analysis of trabecular architecture and bone remodeling parameters including trabecular number (Tb.N), trabecular separation (Tb.Sp), trabecular thickness (Tb.Th), and fractal dimension (FD). *N* = 6 (scale bars = 1 mm). Significant differences were presented as *P < 0.05, **P < 0.01, and ***P < 0.001. Data presented as mean ± SD

At three weeks post-injury, microCT analysis revealed architectural differences in bone repair between systemic and local MSC-treated groups (Fig. 7). The difference in bone volume fraction (BV/TV%) did not reach any significant differences between groups (Fig. 7), possibly due to the early time point of characterization. However, the local group demonstrated more mature bone characteristics, including significantly lower bone surface density (BS/TV) and greater anisotropy (DA). The lower BS/TV in the local group may reflect a more consolidated, mature bone formation. The degree of anisotropy (DA) was significantly increased in the local MSC group (*p* < 0.05), suggesting a more aligned trabecular structure, indicating improved mechanical integrity. Other trabecular parameters, including trabecular number (Tb.N) and thickness (Tb.Th), showed no significant differences between groups. Fractal dimension (FD), a measure of architectural complexity, was similar across groups. These findings highlighted that more definitive changes may require a longer healing interval for full characterization.

### Histological analysis reveals greater mineralization and less cartilage in fracture callus with local MSC delivery

**Fig. 8:**
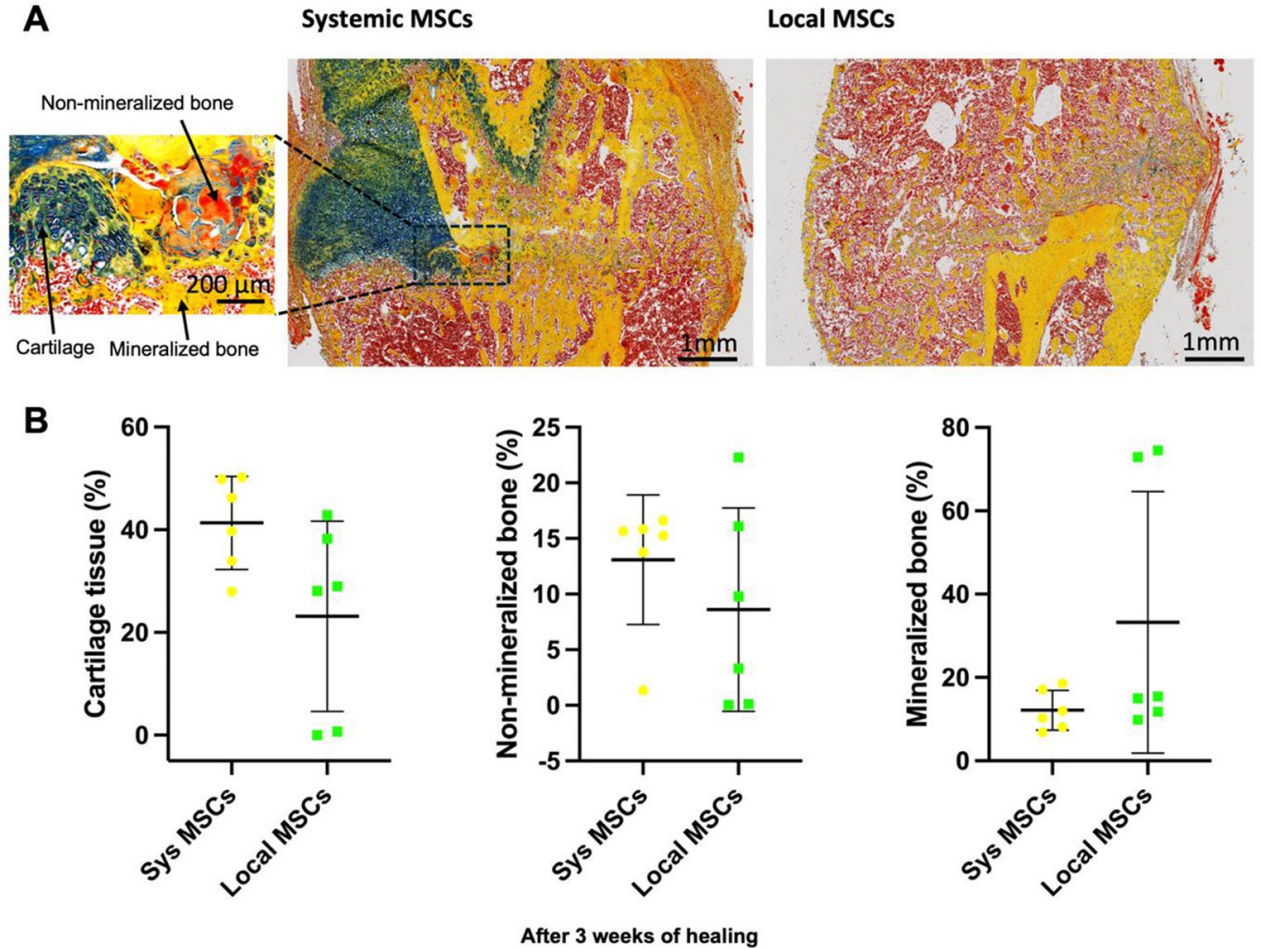
Locally injected mesenchymal stem cells (MSCs) enhance fracture healing in polytrauma after three weeks of healing. (**A**) Representative Movat Pentachrome histology staining for mineralized tissue (yellow), non-mineralized tissue (red), cartilage formation (green), bone marrow (brown) (scale bars = 1 mm) after three weeks of healing. (**B)** Quantitative histological analysis of mineralized and non-mineralized tissue, as well as cartilage formation. *N* = 6. Data presented as mean ± SD

In addition, the histological analysis demonstrated that while systemic MSCs had a higher average of cartilage formation in the fracture callus, the mineralized bone tissue was more prevalent and homogeneous in the locally delivered MSCs fracture callus (Fig. 8). Although quantitative histomorphometry did not reveal statistically significant differences in mineralized or cartilaginous tissue content between the groups, descriptive observations suggested a trend toward more mature bone formation with local MSC therapy.

### Local MSC delivery enhances vascularization, osteogenic activity, and bone remodeling balance

Immunohistochemical analysis at 3 weeks post-injury revealed significant differences between systemic and local MSC delivery groups in markers related to angiogenesis, osteogenesis, and bone remodeling.

**Fig. 9:**
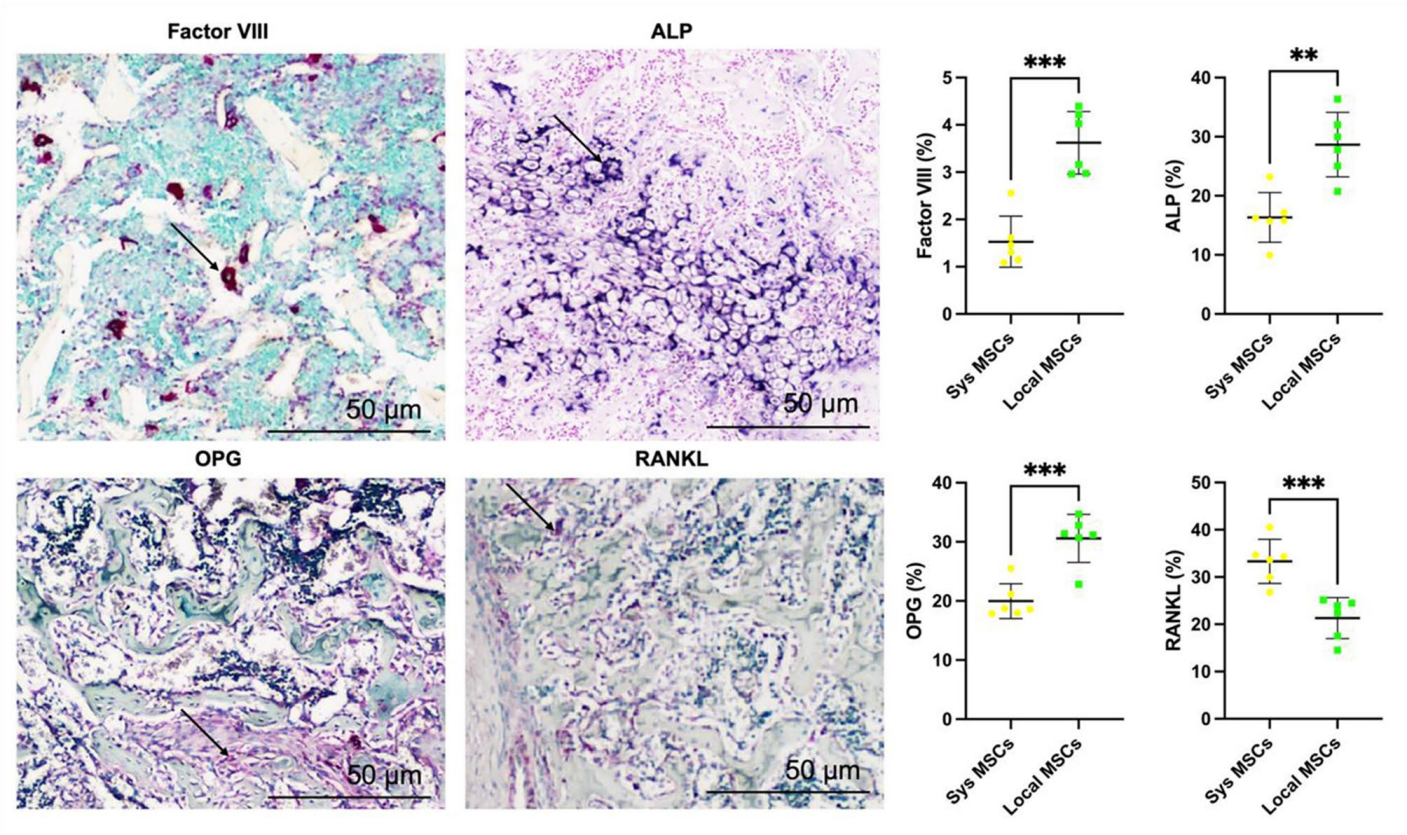
Local MSC delivery enhances angiogenesis, osteogenesis, and bone remodeling signaling at 3 weeks post-injury. Representative immunohistochemical images (form local MSCs group at the fracture site) and quantification of Factor VIII (vascular marker), ALP (osteoblast activity), OPG, and RANKL (bone remodeling regulators) in fracture callus tissues from polytrauma rats treated with either systemic (Sys MSCs) or local (Local MSCs) MSC delivery. Local MSC treatment significantly increased vascular density (Factor VIII) and osteogenic activity (ALP); and shifted the bone remodeling balance toward formation with increased OPG and decreased RANKL expression. Arrows indicate positive staining. Scale bars = 50 μm. Quantification shows percentage of positively stained area per callus section. *N* = 6. Significant differences were presented as *P < 0.05, **P < 0.01, and ***P < 0.001. Data presented as mean ± SD

Factor VIII staining, a marker of endothelial cells and neovascularization, showed increased vascular density in the HA + MSC local group compared to the systemic group (Fig. 9). Quantitative analysis revealed a significantly higher percentage of Factor VIII-positive area in the local MSC group (***p < 0.001), indicating enhanced angiogenesis at the fracture site.

Alkaline phosphatase (ALP) staining, indicative of early osteogenic activity, was more prominently expressed in the local MSC group, with stronger and more widespread staining in the developing callus. Quantification confirmed significantly elevated ALP-positive areas in the local group (**p < 0.01), suggesting accelerated osteoblastic activity (Fig. 9).

Bone remodeling markers demonstrated a favorable osteogenic balance in the local MSC delivery group. Osteoprotegerin (OPG), a decoy receptor that inhibits osteoclastogenesis, was significantly higher in the local group (***p < 0.001), while RANKL expression, which promotes osteoclast differentiation, was significantly lower (***p < 0.001). The resulting shift toward a higher OPG/RANKL ratio supports a remodeling environment more conducive to bone formation and less prone to resorption (Fig. 9). These findings highlight the advantage of local MSC delivery in promoting early vascularization, osteogenesis, and favorable bone remodeling signaling during the early phase of fracture healing in a polytrauma model.

## Discussion

Our study highlights the distinct inflammatory profiles associated with isolated fractures versus polytrauma. In vitro secretome analysis revealed that MSCs exposed to polytrauma serum produced significantly elevated levels of pro-inflammatory cytokines, including IL-6, IL-1α, IL-1β, TNF-α, IFN-γ, and GM-CSF, relative to fracture serum. These cytokines, alongside chemokines such as MCP-1, MCP-5, MIP-1α, and RANTES/CCL5, are key mediators of sustained immune activation and cell recruitment to sites of injury. This pattern is consistent with previously described damage-associated molecular patterns (DAMPs), which trigger systemic inflammatory responses following trauma [27–29]. If unchecked, this systemic inflammation can escalate into systemic inflammatory response syndrome (SIRS) and potentially multiple organ dysfunction syndrome (MODS).

By day 7, the inflammatory signature in the polytrauma group remained elevated, particularly IL-12p70 and IL-17, both associated with chronic inflammation and impaired healing [23, 24, 26]. Additionally, the marked rise in CXCL15 (lungkine), a chemokine involved in neutrophil recruitment, supports the role of pulmonary trauma in sustaining systemic inflammation [30]. In contrast, MSCs cultured with fracture serum exhibited a more balanced secretome profile, characterized by increased IL-10 and pro-angiogenic factors such as VEGF-R1, suggesting an environment favorable for vascularization and tissue regeneration.

Our in vivo data further confirmed that polytrauma induces a sustained systemic inflammatory response. Serum cytokine levels of IL-23, IL-1β, TNF-α, and IFN-γ remained significantly elevated at weeks 1 and 3 post-injury, reinforcing the notion that polytrauma disrupts the normal progression from inflammation to repair. These observations align with our previous findings that thoracic trauma prolongs the inflammatory phase of fracture healing [2], as well as conclusions by Mangum et al. [4], who showed that burn and thoracic trauma impair healing via systemic inflammatory mechanisms.

Both systemic and local MSC therapies significantly reduced these inflammatory markers, but local delivery showed greater suppression, particularly of IL-23 and TNF-α. Moreover, local MSC treatment more effectively reduced IL-12p70, implicated in persistent Th1-mediated inflammation and fracture nonunion [31]. Systemic MSC administration also conferred reductions in IFN-γ and IFN-β, but with less consistency, likely due to pulmonary entrapment of MSCs following intravenous delivery, as supported by our imaging data. This is consistent with reports that intravenously delivered MSCs are sequestered in the lungs due to their size and integrin expression profiles [7, 8] limiting their availability at the fracture site and reducing therapeutic efficacy.

We observed that local MSC delivery reduced variability in inflammatory responses within the polytrauma group. This effect is likely due to the higher retention and site-specific activity of MSCs at the fracture site, minimizing systemic dilution and providing more consistent immunomodulation. This is particularly relevant in polytrauma, where systemic inflammation can impair both MSC survival and homing.

These inflammatory cytokines, particularly IL-6, TNF-α, IL-17 A, and IL-23, are not only markers of systemic inflammation but active participants in impairing fracture healing through their influence on bone remodeling pathways. They interact with key signaling axes such as NF-κB, RANKL/OPG, and JAK/STAT. For instance, TNF-α and IL-6 have been shown to activate NF-κB and STAT3 signaling, promoting osteoclastogenesis by upregulating RANKL expression while simultaneously inhibiting osteoblast function [32–35]. IL-17 A and IL-23 further compound this effect by dampening MSC osteogenesis and driving inflammatory osteoclast activity through MAPK and NF-κB pathways [36–38]. Additionally, dysregulation of the RANKL/OPG balance, where osteoblast-derived RANKL promotes osteoclast maturation unless counteracted by OPG, has been directly linked to impaired callus formation and excessive resorption in trauma models [39]. This mechanistic interplay underscores the importance of therapies that can interrupt this pathological feedback loop and re-establish a regenerative microenvironment.

IVIS imaging confirmed the pulmonary entrapment of systemically administered MSCs, with no detectable migration to the fracture site. This phenomenon, attributed to the MSCs’ size and adhesion profile, has been documented previously [7]. The lack of signal at the fracture site further underscores the limitations of systemic delivery, especially in trauma models with lung involvement [8]. In contrast, MSCs delivered within HA hydrogels remained localized to the fracture, enabling sustained paracrine signaling within the injury microenvironment.

MicroCT data showed that local MSC therapy was associated with improved trabecular architecture. While no statistical significance was observed for BV/TV, the differences in remodeling metrics (e.g., anisotropy and surface density) suggested a more organized bone architecture in the local MSC group. These observations should be interpreted cautiously, given the relatively short 3-week healing window. Our histological findings showed increased mineralized bone and reduced cartilage in the local MSC group, indicating a more advanced state of endochondral ossification. This was consistent with our immunohistochemical analysis that demonstrated enhanced angiogenesis and osteogenic signaling in the local MSC group. Specifically, increased Factor VIII and ALP expression indicate improved vascularization and osteogenic activity, respectively. Additionally, the favorable shift in the OPG/RANKL axis toward bone formation suggests that local MSC delivery promoted a remodeling environment conducive to fracture repair. These localized effects reinforce the therapeutic potential of direct MSC administration in polytrauma settings, where rapid tissue regeneration and remodeling are essential.

The apparent discrepancy between the in vitro and in vivo effects of MSCs can be explained by the absence of immune cell interactions in vitro. In vivo, local MSC delivery allows for direct engagement with host immune cells, particularly macrophages, in the fracture microenvironment. This interaction plays a critical role in modulating inflammation and promoting bone repair. MSCs are known to drive the phenotypic shift from pro-inflammatory M1 to anti-inflammatory M2 macrophages, a process essential for progression from inflammation to tissue regeneration [40]. Emerging evidence suggests that both M1 and M2 macrophages can influence MSC osteogenic activity, albeit at different stages of bone healing [40]. M1 macrophages may enhance early osteogenesis through elevated PGE2 and oncostatin M signaling, while M2 macrophages are associated with increased matrix mineralization and higher BMP-2 production [41, 42]. Additionally, macrophage-MSC crosstalk is influenced by factors such as MSC age and co-culture conditions, highlighting the complexity of their interplay [40]. These interactions are likely to be pivotal in the superior regenerative outcomes observed with local MSC delivery in our model, as opposed to the more inflammatory response seen in vitro under serum-only exposure [40].

Several limitations should be acknowledged. First, our ability to track MSCs was restricted to a 1-week timepoint due to limited luciferase signal at day 3 and week 3. This limitation may reflect the known transient viability of MSCs post-transplantation. Previous work by Pang et al. [43] has emphasized that MSCs exert therapeutic effects largely through apoptosis-driven immunomodulation, rather than engraftment or differentiation. This aligns with our findings that MSCs were undetectable beyond week 1, yet their early presence correlated with systemic cytokine modulation and changes in fracture healing. Second, our study used only young male mice. While this aligns with typical demographics in polytrauma, age and sex can affect immune responses and fracture healing. These variables should be explored in future work. Additionally, we did not perform mechanical testing, focusing instead on microCT and histology. Including mechanical outcomes in future studies would further validate the functional significance of observed bone remodeling. A recent study by Huang et al. [44] reported that locally injected MSCs remained at the fracture site for up to two weeks, whereas systemically injected MSCs first accumulated in the lungs and then migrated to the fracture over 8–9 days. This suggests that timing and cell trafficking dynamics should be considered when interpreting imaging data and therapeutic effects. A key limitation of this study is the 3-week follow-up, which primarily reflects the early stages of fracture healing, including inflammation and endochondral ossification. As such, we cannot draw conclusions regarding the quality or strength of the fully remodeled bone. Future studies incorporating extended time points (at least for 3 and 6 months) will be necessary to determine whether the observed early improvements translate to long-term functional recovery.

Looking ahead, given that excessive inflammation hinders healing, co-delivery of MSCs with anti-inflammatory agents could further improve outcomes in polytrauma-associated fractures. Additionally, future work should explore the role of sex, aging, and long-term healing timelines in evaluating MSC-based therapies. Our HA-based hydrogel system played a role in facilitating this localized therapeutic effect. HA provided a supportive, biocompatible matrix for MSC retention and delivery. However, despite promising trends, neither systemic nor local MSC delivery resulted in complete fracture healing within three weeks. To further improve osteogenesis, we plan to augment our HA-MSC platform with osteoinductive factors such as disordered peptides, aiming to accelerate healing and restore bone integrity more rapidly.

## Conclusions

This study demonstrated that polytrauma, modeled by combined chest injury and femur fracture, induces a pathologically heightened and prolonged inflammatory response that impairs fracture healing. This was evidenced by sustained elevations of pro-inflammatory cytokines and chemokines in serum, which were associated with delayed tissue repair and increased variability in healing outcomes. We also showed that systemic mesenchymal stromal cell (MSC) delivery is limited by pulmonary entrapment, significantly reducing MSC availability at the fracture site and diminishing therapeutic efficacy. In contrast, local MSC delivery effectively modulated local inflammation, suppressed systemic cytokine levels, and promoted more consistent and enhanced fracture healing and angiogenesis. IVIS imaging confirmed that systemically administered MSCs accumulated in the lungs, while locally delivered MSCs remained at the fracture site, supporting the superiority of localized therapy in polytrauma settings. Our findings provide support for using biomaterial-encapsulated local MSC delivery to improve angiogenesis and early osteogenesis outcomes in complex injury models. Future research should focus on optimizing the immunomodulatory properties of MSC-laden hydrogels through the incorporation of osteogenic cues and tailoring biomaterial properties. Additionally, exploring the influence of aging and sex differences on MSC function will be crucial for developing more personalized regenerative therapies.

## Supplementary Information

Below is the link to the electronic supplementary material.

Supplementary Material 1

## Data Availability

The datasets used and/or analyzed during the current study are available from the corresponding author on reasonable request.

## Abbreviations

MSCs: Mesenchymal stem cells
IL-1 β: Interleukin 1 beta
TNF-α: Tumor necrosis factor-alpha
IFN-γ: Interferon-gamma
MCP: Monocyte chemoattractant protein
BMPs: Bone morphogenetic proteins
DBM: Demineralized-based matrix
HA: Hyaluronic acid
ECM: Extracellular matrix
ROI: Region of interest
BV: Bone volume
TV: Total volume
BS/TV: Bone surface density
Tb.Th: Trabecular thickness
Tb.Sp: Trabecular separation
VCAM-1: Vascular cell adhesion molecule-1
VEGF-R1: Vascular endothelial growth factor receptor 1
sTNF-R: TNF-α soluble receptors
