# Supplementary material for: Systemic versus local delivery of mesenchymal stem cells to improve the early stages of fracture healing in a polytrauma model
Source: J Biol Eng. 2025 Sep 30;19:82. doi: 10.1186/s13036-025-00554-4 (PMC12487495; doi:10.1186/s13036-025-00554-4)
Supplement: Supplementary file 1 — Supplementary Material 1 [file 13036_2025_554_MOESM1_ESM.docx]

**Systemic versus Local Delivery of Mesenchymal Stem Cells to Improve the Early Stages of Fracture Healing in A Polytrauma Model**

*Augustine Mark Saiz^1,2^*, Maryam Rahmati^1,2^, Soren David Johnson^1^, Aneesh Satish Bhat^1^, Tony Daniel Baldini^1^, Øystein Øvrebø^2^,* Liebert Parreiras Nogueira^2,3^, *Thaqif El Khassawna^4,5^, Sabine Stötzel^4^, Fernando A. Fierro^6,7^, Mark A. Lee^1^, J. Kent Leach^1^, Håvard Jostein Haugen^2^*

*^1^Department of Orthopaedic Surgery, UC Davis Health, 4860 Y Street, Suite 3800, Sacramento, CA, 95817, USA*

*^2^Department of Biomaterials, Institute of Clinical Dentistry, University of Oslo, 0318 Oslo, Norway*

*^3^Oral Research Laboratory, Institute of Clinical Dentistry, University of Oslo, 0318 Oslo, Norway*

*^4^Experimental Trauma Surgery, Justus-Liebig University Giessen, Giessen, Germany*

*^5^Faculty of Pharmacy, University of Jordan, Amman, Jordan*

*^6^Institute for Regenerative Cures, University of California Davis, 2921 Stockton Blvd. Room 1300, Sacramento, CA, 95817, USA*

*^7^Department of Cell Biology and Human Anatomy, University of California Davis, Davis, USA*

*^*^Email of the Corresponding Author:* [*amsaiz@health.ucdavis.edu*](mailto:amsaiz@ucdavis.edu)

**Material Characterization**

As previously reported (1): Amplitude-sweep rheometer at 25 °C (0.01–1400 % strain, 10 rad s⁻¹) revealed a broad linear-viscoelastic domain in which the storage modulus (G′) remained essentially constant at ≈ 75 Pa until ∼100 % strain, after which G′ converged with the loss modulus (flow point at ≈10³ % strain) to signify the transition to viscous behavior. Frequency sweeps (0.1–40 rad s⁻¹, 1 % strain) were frequency-independent, confirming a well-cross-linked elastic network. Real-time monitoring of in-situ gelation at 40 °C under 0.1 % strain showed that G′ overtook G″ after roughly 60 min and plateaued at ≈ 1 kPa. Chemical cross-link formation was validated by FTIR, which displayed characteristic ether stretches at 1050–1150 cm^-1^ and carboxylate absorption at 1650 cm⁻¹, while the near absence of epoxy-end signals at 2900–3000 cm^-1^ indicated efficient removal of unreacted BDDE. 1^H^/13^C^ NMR spectroscopy of swollen gels yielded a degree of modification of 12 % and an effective cross-linking ratio of 0.24. Hydration studies demonstrated rapid water uptake, with samples swelling to 135 ± 7 % of their initial mass within 1 h and 596 ± 18 % after 24 h in distilled water. Environmental-SEM of dehydrated cross-sections showed a heterogeneous pore architecture comprising circular voids of approximately 20–200 µm interspersed with sheet-like domains. Leachable analysis by 1^H^ NMR of dialysis media confirmed that residual BDDE was below the detection threshold after 18 h in distilled water. ISO 10993-5 cytotoxicity assays using MC3T3-E1 pre-osteoblasts recorded < 30 % LDH release and > 90 % CCK-8 viability, classifying the material as non-cytotoxic. These data establish HA-BDDE as an injectable, weakly elastic (G′ ≈ 1 kPa post-gelation), a highly hydrated hydrogel with defined cross-link density and negligible leachable. All manufacturing steps were carried out in accordance with Good Laboratory Practice (GLP) within an ISO 13485-2016 certified facility as previously described (1).


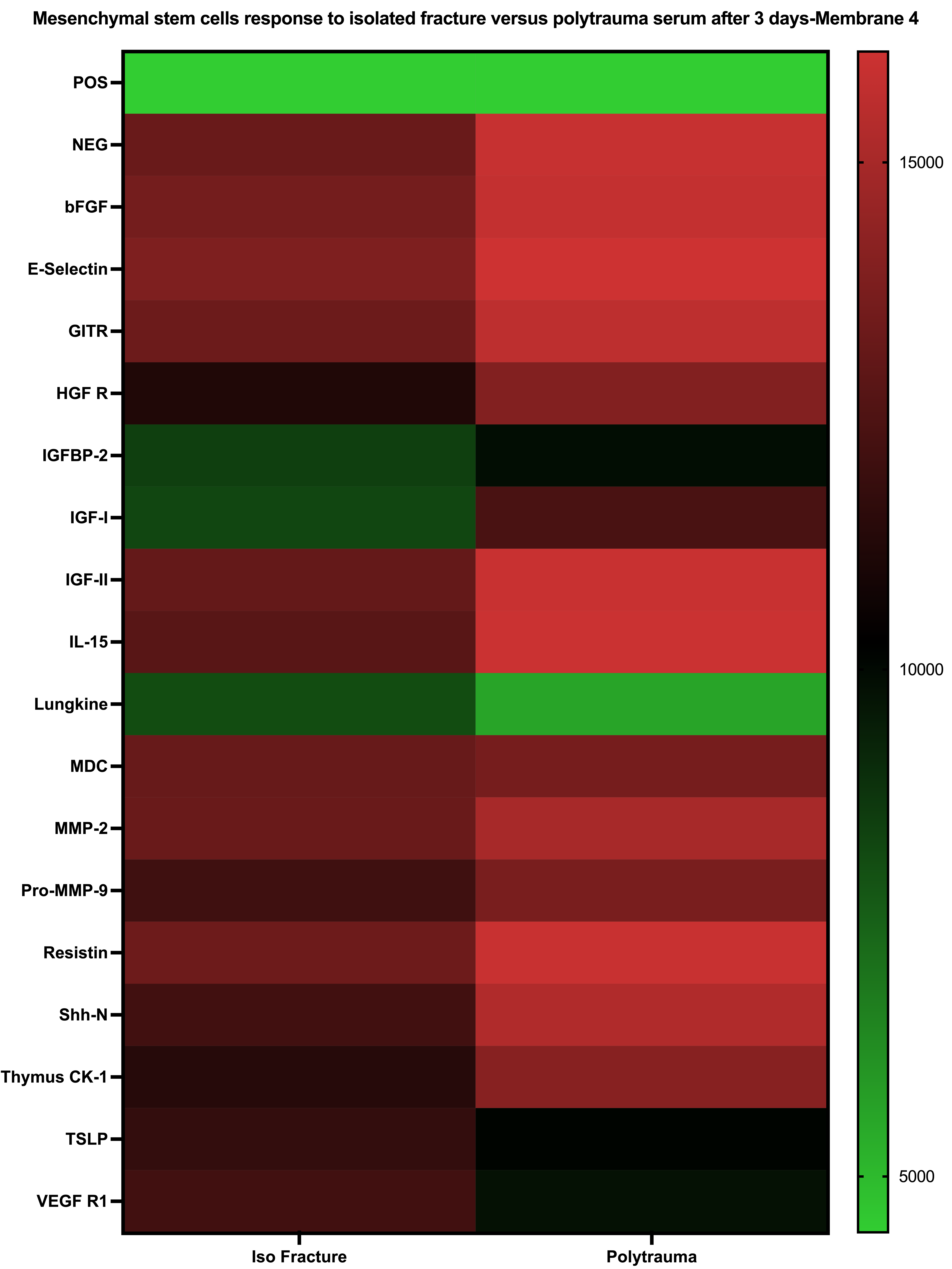


**Supp Figure 1.** Polytrauma serum induces higher proinflammatory responses in mesenchymal stem cells (MSCs). Further secretome profiling of mesenchymal stem cells (MSCs) pre-exposed to isolated fracture versus polytrauma serum after 3 days of culturing in osteogenic media. Cytokine levels were quantified, highlighting differential inflammatory responses between the two conditions. N=8 samples pooled together for each group. Green color depicts low levels and red depicts high levels of expression. POS= positive control spots, which was used as the internal control/baseline.


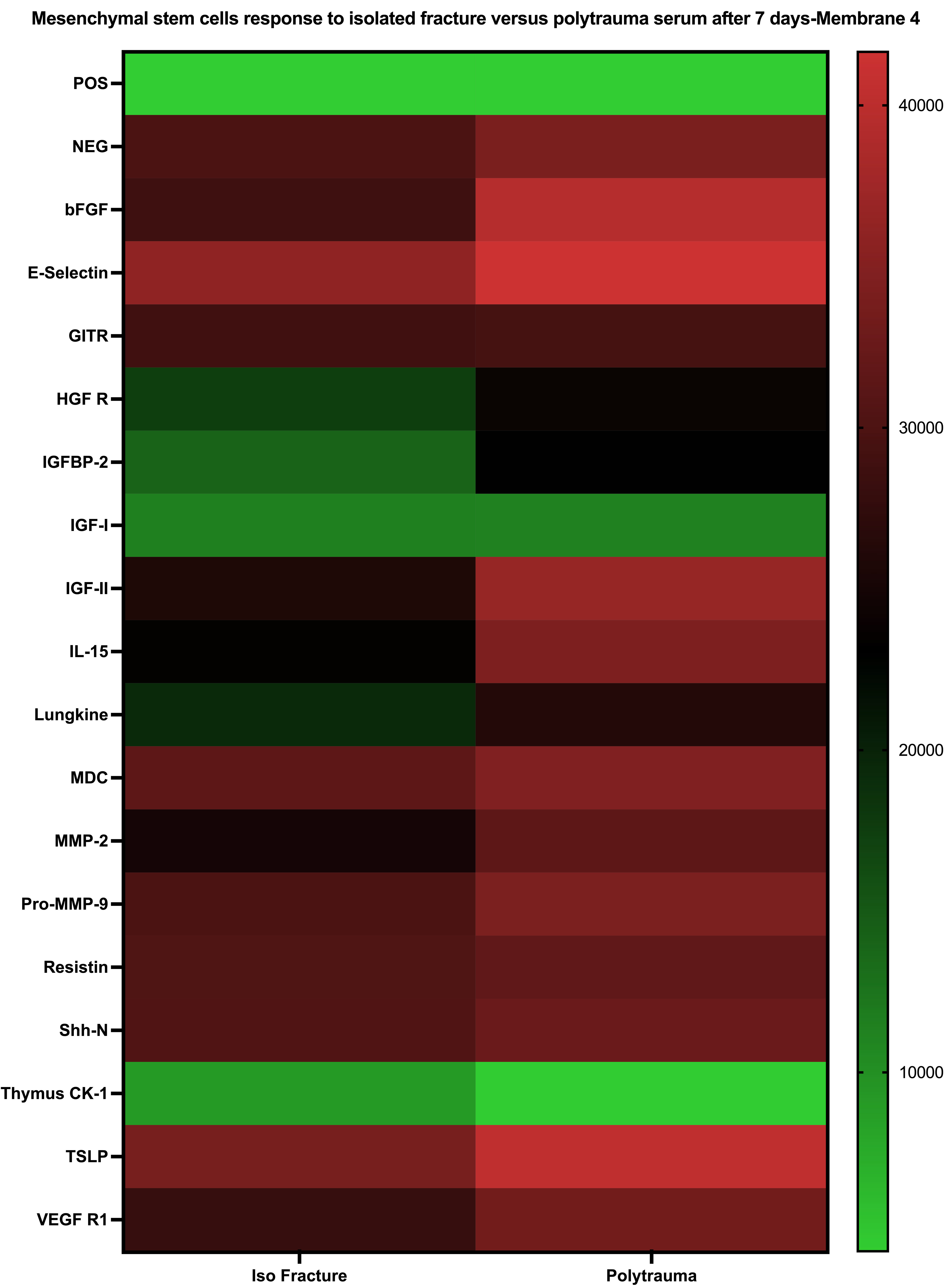


**Supp Figure 2.** Polytrauma serum induces higher proinflammatory responses in mesenchymal stem cells (MSCs). Further secretome profiling of MSCs pre-exposed to isolated fracture versus polytrauma serum after 7 days of culturing in osteogenic media. Cytokine levels were quantified, highlighting differential inflammatory responses between the two conditions. N=8 samples pooled together for each group. Green color depicts low levels and red depicts high levels of expression. POS= positive control spots, which was used as the internal control/baseline.

**References**

1. Øvrebø Ø, Giorgi Z, De Lauretis A, Vanoli V, Castiglione F, Briatico-Vangosa F, et al. Characterisation and biocompatibility of crosslinked hyaluronic acid with BDDE and PEGDE for clinical applications. React Funct Polym. 2024;200.
